# Human Melioidosis Caused by Novel Transmission of *Burkholderia pseudomallei* from Freshwater Home Aquarium, United States^1^

**DOI:** 10.3201/eid2712.211756

**Published:** 2021-12

**Authors:** Patrick Dawson, Monique M. Duwell, Mindy G. Elrod, Ruth J. Thompson, David A. Crum, Ruth M. Jacobs, Jay E. Gee, Cari B. Kolton, Lindy Liu, David D. Blaney, LaToya Griffin Thomas, Denise Sockwell, Zachary Weiner, William A. Bower, Alex R. Hoffmaster, Johanna S. Salzer

**Affiliations:** Centers for Disease Control and Prevention, Atlanta, Georgia, USA (P. Dawson, M.G. Elrod, J.E. Gee, C.B. Kolton, L. Liu, D.D. Blaney, Z. Weiner, W.A. Bower, A.R. Hoffmaster, J.S. Salzer);; Maryland Department of Health, Baltimore, Maryland, USA (M.M. Duwell, R.J. Thompson, D.A. Crum);; Holy Cross Germantown Hospital, Germantown, Maryland, USA (R.M. Jacobs);; Virginia Department of General Services, Richmond, Virginia, USA (L. Griffin Thomas);; Virginia Department of Health, Richmond (D. Sockwell)

**Keywords:** melioidosis, *Burkholderia pseudomallei*, bacteria, One Health, transmission, freshwater home aquarium, human infection, imported tropical fish, pets, whole-genome sequencing, zoonoses, Southeast Asia, Maryland, United States

## Abstract

Nearly all cases of melioidosis in the continental United States are related to international travel to areas to which *Burkholderia pseudomallei*, the bacterium that causes melioidosis, is endemic. We report the diagnosis and clinical course of melioidosis in a patient from the United States who had no international travel history and the public health investigation to determine the source of exposure. We tested environmental samples collected from the patient’s home for *B. pseudomallei* by PCR and culture. Whole-genome sequencing was conducted on PCR-positive environmental samples, and results were compared with sequences from the patient’s clinical specimen. Three PCR-positive environmental samples, all collected from a freshwater home aquarium that had contained imported tropical fish, were a genetic match to the clinical isolate from the patient. This finding suggests a novel route of exposure and a potential for importation of *B. pseudomallei*, a select agent, into the United States from disease-endemic areas.

Melioidosis is a severe, potentially life-threatening bacterial disease caused by *Burkholderia pseudomallei*, a gram-negative bacterium found in water and soil in tropical and subtropical environments worldwide (*1*). Melioidosis might manifest as localized, pulmonary, systemic, or disseminated infections. However, melioidosis symptoms are nonspecific, and it is often misdiagnosed (*1*,*2*). Exposure to *B. pseudomallei* occurs through inhalation of contaminated dust or water droplets, ingestion of contaminated water, and direct contact with contaminated water or soil, particularly through cuts or abrasions (*3*). The incubation period in acute cases ranges from 1 to 21 days, although activation of latent infections might develop many years later (*4*,*5*). Persons at greater risk for developing of melioidosis include those with diabetes, liver disease, renal disease, chronic lung disease, thalassemia, cancer, and other immunocompromising conditions (*6*,*7*).

Melioidosis (formerly Whitmore’s disease) was described in 1912 (*8*), and cases were historically identified primarily in northern Australia and areas of Southeast Asia, such as Thailand, where melioidosis is hyperendemic (*9*). However, the known geographic range has expanded considerably in recent decades; the estimated global burden is 165,000 human cases/year (*10*). On the basis of clinical cases or environmental isolation, *B. pseudomallei* is now suspected to be endemic to the environment in parts of Central and South America, the Caribbean, Mexico, and potentially in areas of the continental United States, such as Texas (*11*–*14*). Despite increased recognition of the expansive range of *B. pseudomallei*, nearly all US cases are related to previous residence in or travel to disease-endemic areas outside the continental United States (*15*).

## Case Report

In October 2019, the US Centers for Disease Control and Prevention (CDC) was notified by the Maryland Department of Health and through the CDC Laboratory Response Network (LRN) of a preliminary positive bacterial isolate of *B. pseudomallei* from a blood culture specimen from a Maryland resident. The clinical specimen had been forwarded to the Virginia Division of Consolidated Laboratory Services, where it was confirmed, and shipped to CDC for simultaneous confirmation and genomic analysis.

The patient, a 56-year-old woman, was hospitalized on September 20, 2019 (day 0; Figure), because of fever, cough, and chest pain with onset 2 days earlier. The patient had a history of polymyositis, rheumatoid arthritis, and diabetes mellitus, and reported to have stopped long-term immunosuppressive medications (methotrexate, azathioprine, and prednisone) 1 month before symptom onset.

A thoracic radiograph on day 0 showed a right perihilar and lower lobe infiltrate, consistent with pneumonia. A noncontrast computed tomography (CT) scan on day 3 showed air space consolidation in the right lower lobe consistent with pneumonia, additional bibasilar air space densities, and a small right pleural effusion. Other notable clinical laboratory results at presentation included an increased leukocyte count of 22,800 cells/μL (reference range 4,500‒11,000 cells/μL) and a decreased sodium level of 125 mmol/L (reference range 135‒145 mmol/L).

We obtained blood cultures on day 0 before administration of antimicrobial drugs. Gram-negative rods were identified in 4 initial blood cultures; the rods were subsequently identified as *B. pseudomallei*. Three additional blood cultures obtained on days 2–4 grew the same organism. The patient was given ceftriaxone and azithromycin on days 0–3, then on day 4, treatment was changed to meropenem after *B. pseudomallei* was identified. The patient showed gradual symptom improvement; fever resolved, and leukocyte count normalized. The intensive phase duration was extended because of persistent bacteremia. She was discharged on day 11 and continued taking intravenous meropenem as an outpatient. Antimicrobial drug susceptibility testing was not performed because the patient had been responding to treatment with meropenem before LRN confirmation of *B. pseudomallei*.

After 3 weeks of taking meropenem, the patient’s leukocyte count increased to 14,700 cells/μL, and fever developed (temperature 100.1°F [37.8°C]). A repeat CT scan showed a patchy opacity at the right lung base and bilateral interstitial process consistent with pneumonia, but a 45 × 24 mm mass in the right lung posteriorly could not be excluded and increased concern for pulmonary abscess. The patient was readmitted, sulfamethoxazole/trimethoprim was given, and treatment with meropenem continued. She clinically improved and was discharged after 1 week. Repeat, noncontrast, thoracic CT scans showed improvement of right basilar consolidation after 6 weeks of meropenem and sulfamethoxazole/trimethoprim and resolution after 10 weeks. The patient completed eradication therapy with a 10-week course of meropenem and 12-week course of sulfamethoxazole/trimethoprim.

## Methods

### Case Investigation

We conducted an initial epidemiologic investigation to assess the travel history of the patient and other possible exposures to *B. pseudomallei*. Interviews were conducted October–December 2019, with the patient and other household members. After establishing no international travel history and identification of positive environmental samples, the interviews focused on the freshwater home aquariums, tropical fish, and contact with the fish and aquariums of the patient.

### Environmental Sampling

During November 2019, the investigation team visited the home of the patient to collect environmental samples and assess potential sources of *B. pseudomallei*. The patient had 2 freshwater aquariums (tanks A and B, both ≈5 liters). We collected bulk water samples (≈50 mL) from each aquarium and swab specimens of biofilms (3 from tank A and 2 from tank B). We collected an additional 16 samples, including swab specimens of all household faucets, soil from potted plants and around the property, 2 beauty products, and 4 liquid vaping products. During December 2019, the team collected 6 additional specimens: bulk water from tanks A and B, gravel from tank B, filters from tank B, dead fish carcasses from tank B, and a dry artificial plant that had been in tank B but was removed during August 2019. During the second visit, the Maryland Department of Health also performed decontamination, removal, and safe disposal of fish tank B from the home of the patient.

### Laboratory Confirmation

The clinical isolate was confirmed as *B. pseudomallei* at the Virginia Division of Consolidated Laboratory Services and CDC by using the LRN algorithm, including biochemicals and PCR. All 29 environmental samples were sent to CDC for culture and identification. We directly inoculated all environmental samples into TBSS-C50 (Galimand) broth and incubated broths in a shaking incubator at 37ºC for 6 days. We then cultured enriched broths onto Ashdown agar, and extracted DNA by using a QIAamp Fast Stool Mini Kit (QIAGEN, https://www.qiagen.com) or testing by real-time PCR. Suspected colonies from Ashdown agar were selected for further workup, and confirmation of isolates followed the LRN algorithm.

### Multilocus Sequence Typing and Whole-Genome Sequencing

CDC performed multilocus sequence typing on isolate MD2019a (clinical). CDC also performed whole-genome sequencing (WGS) for isolates MD2019a (clinical), MD2019b (water from tank B collected during November 2019), and MD2019c (swab specimen of biofilms from tank B collected during November 2019) by using the Nextera FLEX Kit (https://www.illumina.com) for library preparation and the iSeq 100 instrument (Illumina) with a 2 × 151-bp kit. We analyzed draft genomes along with a reference panel of *B. pseudomallei* genomes from publicly available sources representing geographic diversity by using Parsnp in the HARVEST 1.3 suite (https://harvest.readthedocs.io/en/latest/content/parsnp.html) to detect single-nucleotide polymorphisms (SNPs).

## Results

### Initial Investigation

The initial epidemiologic investigation showed that the patient was a long-time resident of Maryland who had never traveled outside the continental United States. She reported previous pet ownership of reptiles and cats >5 years ago. She denied any other direct soil or environmental water exposure. She also denied use of herbal products or products known to be imported from Asia or Australia. She reported use of vaping products. No family members or close contacts had an illness similar to that of the patient.

Multilocus sequence typing yielded sequence type 369, which had been seen in examples from Malaysia, Thailand, and Vietnam (*16*). WGS of the clinical isolate, MD2019a, showed that when compared to a panel of publicly available genomes composed to represent geographic diversity, it clustered with genomes from Southeast Asia predominantly associated with Singapore and Malaysia (Appendix Figure). The genetic link of the isolate to Southeast Asia, coupled with lack of international travel history for the patient, led the investigation team to conduct follow-up interviews and environmental sampling in the home of the patient.

### Isolation of *B. pseudomallei* from Environmental Samples

Of the 23 environmental samples collected during November 2019, a total of 3 samples, all from tank B, were positive for *B. pseudomallei* by culture and real-time PCR. All other November 2019 samples were negative. The water and gravel samples from tank B collected during December 2019 were also positive for *B. pseudomallei* by PCR and culture. The artificial plant that was removed from the tank during August 2019 was positive by PCR, but no growth was observed in culture. The December 2019 water sample from tank A was negative.

### Genetic Match of Aquarium Isolates with the Clinical Isolate

Comparison of the draft genome sequences between the clinical isolate (MD2019a) and the 2 aquarium samples from tank B (MD2019b and MD2019c) showed no SNPs between MD2019a and MD2019c, and only 1 SNP was detected between MD2019a and MD2019b. This finding indicates that all 3 isolates were clonal.

### Follow-Up Patient Interviews

The patient reported she had purchased fish, aquariums, and associated supplies during July 2019 (Figure). She purchased the tanks and gravel substrate at a large retail store. She purchased 3 cherry barbs (*Puntius titteya*) for tank A from a retail pet store, and all were alive at the time of her interview. She purchased 3 fancy-tailed guppies (*Poecilia reticulata*) for tank B from the same store, and they died during August 2019. During October 2019, after her illness onset, she purchased 3 tiger barbs (*Puntigrus tetrazona*) for tank B from the same store, and they died during November 2019.

**Figure:**
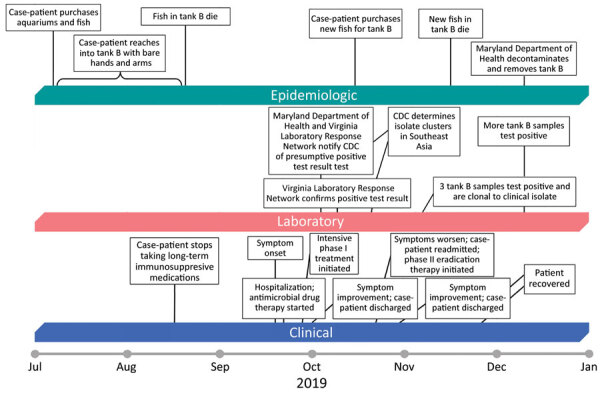
Clinical, laboratory, and epidemiologic timeline for a patient who had melioidosis, Maryland, USA, 2019. CDC, Centers for Disease Control and Prevention.

The patient reported that the water in tank B was persistently cloudier than the water in tank A and more difficult to keep clean. She reported that she was the primary caretaker of the fish and recalled reaching bare hands and arms into the water and gravel of tank B to disrupt sediment while cleaning it, as recently as August 2019.

## Discussion

This investigation of a patient who had melioidosis and no history of travel to disease-endemic areas provides strong evidence for documented transmission of *B. pseudomallei* from a freshwater home aquarium to a human. Although this type of transmission has not been described in the literature, contamination of aquarium transport water with *B. pseudomallei* used for freshwater tropical fish originating from Singapore has been reported in France (*17*). Furthermore, freshwater fishing practices have been identified as risk factors in countries to which melioidosis is endemic (*7*,*18*,*19*).

With freshwater aquariums as a newly recognized source of possible transmission of *B. pseudomallei* to humans, further investigations are underway to determine the extent of *B. pseudomallei* contamination at the pet store retailer where the patient purchased the pet fish with accompanying aquarium water and at the vendors that imported and supplied freshwater ornamental fish, aquatic plants, and associated aquarium water to this retail location. Because these vendors might distribute freshwater animals and aquatic plants to pet store retailers throughout the United States, identifying possible source(s) of introduction with *B. pseudomallei* in the supply chain is essential to public health.

Federal regulators classify *B. pseudomallei* as a Tier 1 Select Agent because of its heightened risk for deliberate misuse and major potential for mass casualties, economic disruption, critical infrastructure effects, or damaging public confidence (*20*). The United States is the largest importer of ornamental fish, most of which are freshwater and originate in Southeast Asia (*21*,*22*), where *B. pseudomallei* is widespread in the environment. An estimated 11.5 million US households have pet fish; ≈139 million freshwater fish are owned (*23*,*24*). Determining where in the supply chain introduction of the bacteria might occur can lead to development of enhanced surveillance and mitigation procedures at the critical control points, which might prevent further introductions and spread of the bacteria to retailers and homes of consumers.

To prevent or reduce risk of exposure, particularly among persons who have major risk factors, simple precautions can be taken when handling freshwater fish, snails, aquatic plants, aquariums, or other materials in contact with aquarium water, such as gravel, substrate, decorations, filters, and other equipment. CDC recommends thorough handwashing with soap and water before and after handling or cleaning aquariums and feeding fish, wearing gloves to cover any cuts or wounds in the hand while handling fish or aquariums or allowing wounds to fully heal first, avoiding cleaning fish aquariums if immunocompromised or in areas where immunocompromised persons might be present, and not allowing children <5 years of age to clean fish aquariums (*25*).

This report highlights the essential role of molecular epidemiology in public health investigations of melioidosis cases, which identified the likely geographic origin of the bacteria and prompted a public health response that characterized a novel route of exposure. There is growing evidence that US melioidosis cases are not limited to international travelers, including a 2021 multistate cluster involving an organism that is not clonal to the isolates described here, as determined by WGS (*14*,*26*–*29*).

We urge clinicians in the United States to consider melioidosis in patients who have clinically compatible symptoms and exposure to tropical ornamental fish and freshwater aquariums, particularly if patients are immunocompromised, even though such exposure events might be exceedingly rare and few persons show development of melioidosis after exposure to *B. pseudomallei* (*5*). This organism might be difficult for hospital laboratories to diagnose, and automated identification systems in clinical laboratories can misidentify *B. pseudomallei*, highlighting the need for LRN confirmation (*28*).

Clinicians treating melioidosis should consult established treatment guidelines, which were updated in 2020 (*30*,*31*). Likewise, public health investigators should consider inquiring about pet freshwater fish exposure in patients given a diagnosis of melioidosis who have not traveled to a disease-endemic area or have only traveled to locations inconsistent with the geographic profile of the genome of their isolate.

Appendix Additional information on novel transmission of *Burkholderia pseudomallei* from freshwater home aquarium causing human melioidosis, United States.
