# Supplementary material for: Human Melioidosis Caused by Novel Transmission of Burkholderia pseudomallei from Freshwater Home Aquarium, United States
Source: Emerg Infect Dis. 2021 Dec;27(12):3030–5. doi: 10.3201/eid2712.211756 (PMC8632198; doi:10.3201/eid2712.211756)
Supplement: Appendix — Additional information on novel transmission of Burkholderia pseudomallei from freshwater home aquarium causing human melioidosis, United States. [file 21-1756-Techapp-s1.pdf]

Article DOI: https://doi.org/10.3201/eid2712.211756

Human Melioidosis Caused by Novel Transmission of *Burkholderia pseudomallei* from Freshwater Home Aquarium, United States

Appendix

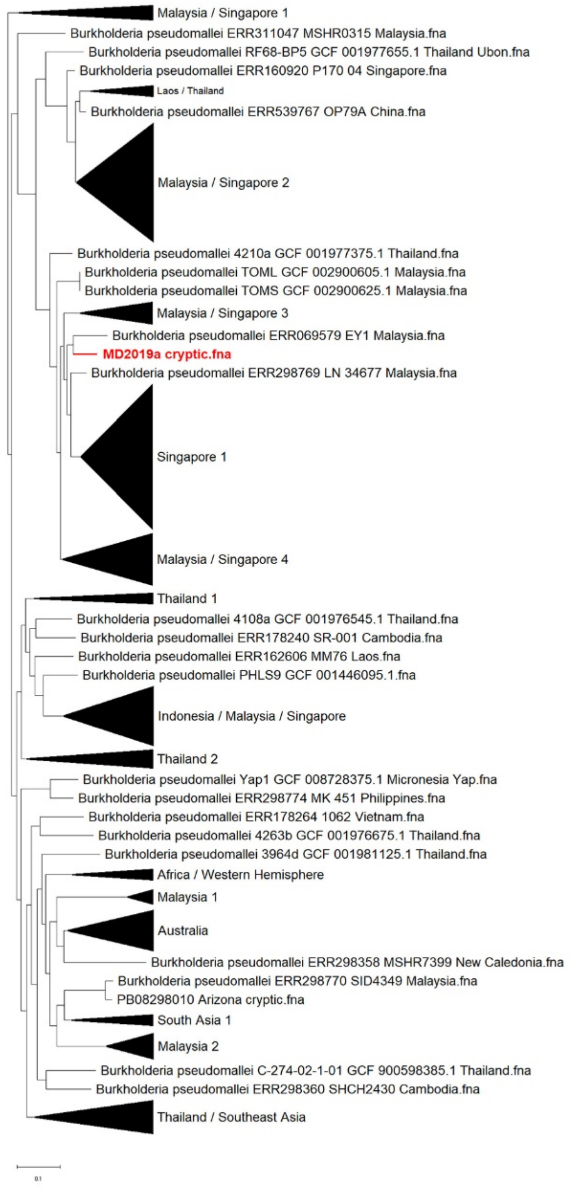

Appendix Figure. Dendrogram of publicly available *Burkholderia pseudomallei* genomes showing the clinical isolate clusters of the patient and isolates from Southeast Asia. The clinical isolate, MD2019a, from the patient is indicated in red. Scale bar indicates nucleotide substitutions per site.
